# A comprehensive toolkit for protein localization and functional analysis in trypanosomatids

**DOI:** 10.1098/rsob.240361

**Published:** 2025-04-02

**Authors:** Athina Paterou, Julia Sáez Conde, Jiří Týč, Jack Daniel Sunter, Sue Vaughan, Keith Gull, Samuel Dean

**Affiliations:** ^1^ Directorate of Biomedical Sciences, Warwick Medical School, University of Warwick, Coventry, UK; ^2^ Department of Biological and Medical Sciences, Oxford Brookes University, Oxford, UK; ^3^ Institute of Parasitology, Biology Centre, Czech Academy of Sciences, České Budějovice, Czech Republic; ^4^ The Sir William Dunn School of Pathology, University of Oxford, Oxford, UK; ^5^ Department of Life Sciences, Imperial College London, London, UK

**Keywords:** toolkit, protein tagging, trypanosomatid, expansion microscopy, trypanosome

## Abstract

African trypanosomes are medically important parasites that cause sleeping sickness in humans and nagana in animals. In addition to their pathogenic role, they have emerged as valuable model organisms for studying fundamental biological processes. Protein tagging is a powerful tool for investigating protein localization and function. In a previous study, we developed two plasmids for rapid and reproducible polymerase chain reaction-based protein tagging in trypanosomes, which enabled the subcellular mapping of 89% of the trypanosome proteome. However, the limited selection of fluorescent protein tags and selectable markers restricted the flexibility of this approach. Here, we present an extended set of >100 plasmids that incorporate universal primer annealing sequences, enabling protein tagging with a range of fluorescent, biochemical and epitope tags, using five different selection markers. We evaluated the suitability of various fluorescent proteins for live and fixed cell imaging, fluorescent movies, and we demonstrate the use of tagging plasmids encoding tandem epitope tags to support expansion microscopy approaches. We show that this series of plasmids is functional in other trypanosomatid parasites, significantly increasing its value. Finally, we developed a new plasmid for tagging glycosylphosphatidylinositol-anchored proteins. We anticipate that this will be an important toolset for investigating trypanosomatid protein localization and function.

**Figure:**
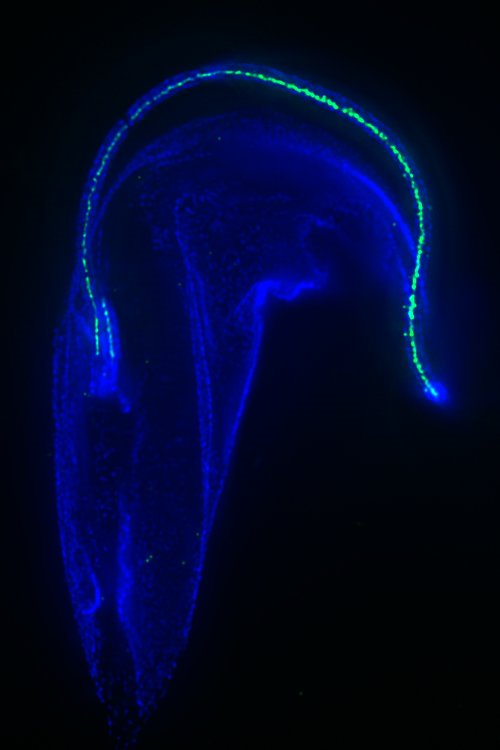

## 1. Introduction

*Trypanosoma brucei* is a protozoan parasite that causes devastating diseases in humans and animals, including sleeping sickness in tsetse endemic Africa. In addition to their pathogenic role, trypanosomes have become valuable model organisms for studying fundamental biological processes due to their extreme and tractable biology, including polycistronic transcription, RNA editing and antigenic variation. Protein tagging is a powerful tool for studying protein localization, interactions and function [1,2]. Previously, a modular plasmid system that comprised two plasmids (pPOTv2 and pPOTv4) was developed for rapid and reproducible protein tagging in trypanosomes [3]. These plasmids utilized a polymerase chain reaction (PCR)-based approach, whereby a drug resistance cassette and protein tag were amplified from the plasmid, with the amplicon targeted for integration into the gene of interest by the long 5′ overhangs on each primer. Although widely used, a more comprehensive toolkit is needed to enable efficient and versatile protein tagging with a variety of fluorescent, epitope and biochemical tags in different trypanosome strains. To address this, we developed a set of more than 100 plasmids with universal primer annealing sequences to enable efficient and versatile protein tagging with a variety of fluorescent and biochemical tags in different trypanosome strains. We evaluated the suitability of different fluorescent proteins, including mNeonGreen [4], mGreenLantern [5] and mScarlet-I [6], for live-cell imaging and determined their brightness and stability under different fixation conditions. We demonstrated the efficacy of a subset of tagging plasmids in several different use cases and in the related parasite, *Leishmania mexicana*. Finally, we have developed an additional tagging plasmid specifically for proteins with signal peptides and/or GPI addition signals. This toolkit will be valuable for investigating protein localization and function in trypanosomatids and will facilitate the study of fundamental biological processes in these important model organisms.

## 2. Results

### 2.1. Design principles and diversity of pPOTv6 and v7 series

**Figure 1.**
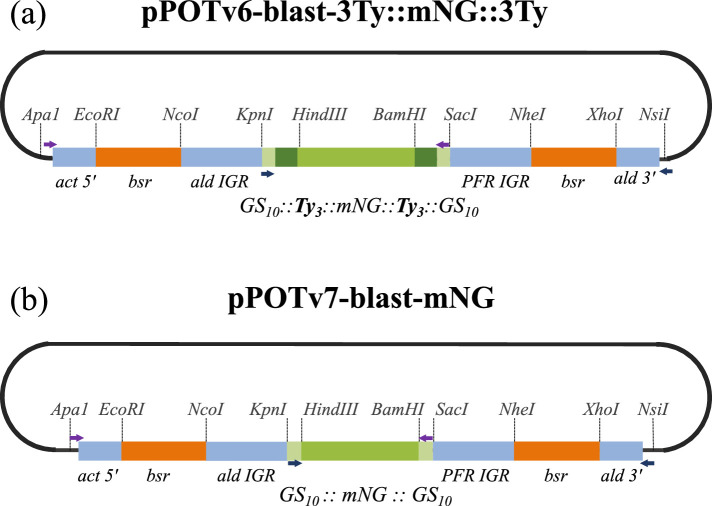
Schematics of pPOTv6 and pPOTv7 design features using (a) pPOTv6-blast-3Ty::mNG::3Ty and (b) pPOTv7-blast-mNG as examples. Modules such as primary and epitope tags, intergenic sequences and drug resistance genes are flanked by unique restriction enzyme sites to facilitate their exchange. Primer annealing sites used to generate N and C terminal tagging amplicons (purple and blue arrows, respectively) are common to all pPOTv6 and pPOTv7 tagging plasmids and are embedded within sequence encoding glycine–serine (GS) linkers and adjacent to the drug resistance regulatory sequence. Note that tagging using pPOTv6 plasmids results in the tagged protein being fused to the primary tag flanked by three tandem copies of an epitope (six total). Act = actin, ald = aldolase, bsr = blasticidin S-resistance gene, IGR = intergenic region, PFR = paraflagellar rod, 5′ = 5′ IGR, 3′ = 3′ IGR.

Here, we describe a new series of PCR-only tagging (pPOT) tagging plasmids with improved design features, including universal primer annealing sequences to enhance flexibility and reduce primer synthesis costs, and the same drug resistance cassette for both amino (N) and carboxyl (C) terminus tagging (figure 1). Additionally, these plasmids have non-chimeric intergenic regulatory sequences to control expression of drug resistance and tagged gene expression, modules flanked by unique restriction enzyme sites to facilitate their exchange and offer five different drug resistance genes for tagging in different trypanosome host strains and for generating complex cell lines. The pPOTv6 plasmids are designed such that the primary tag is flanked by three copies of an epitope tag (Ty1 [7] or Myc [2]) to facilitate western blot analyses, or the 6-His tag to facilitate affinity purification, while the pPOTv7 series plasmids have no flanking epitopes. This new plasmid series encompasses over 20 different fluorescent proteins, three different split fluorescent proteins, three different tags for ‘click’ chemistry, three different proximity labelling tags and several ‘fusion tags’ allowing microscopy to be combined with another assay. In addition, we designed a set of plasmids encoding tandem copies of an epitope tag (including FLAG, HA, Myc, Ty1 and V5 [2]) to support the emerging technique of expansion microscopy [8–10]. A summary of the complete plasmid series is presented in table 1; a comprehensive plasmids list and all plasmids and their GenBank files are available via Addgene (https://www.addgene.org/browse/article/28223366/).

**Table 1.** A summary of pPOTv6 and pPOTv7 plasmids. PCR-only tagging = pPOT. The pPOTv6 and pPOTv7 series encompasses more than 100 different plasmids, incorporating dozens of tags, five different resistance cassettes and several ‘fusion tags’.

type of tag	tag	application
blue fluorescent proteins	BFP, mTFP1, mTurquoise2	live-cell imaging
green fluorescent proteins	mGreenLantern, 3×mNG, mNG, mStayGold[E138D], eGFP, eYFP, moxNG, sfGFP	live-cell imaging
red fluorescent proteins	lss-mOrange, mCherry, mRuby3, mScarlet-I, mScarlet, tagRFPt, tdTomato	live-cell imaging
far-red fluorescent proteins	mCardinal	live-cell imaging
pH sensing fluorescent proteins	pHlourin (M153R), pHlourin2, SypHer2	biological sensors (pH)
click biochemistry	CLIP, SNAP, 3× HaloTag, HaloTag	protein purification, *in vivo* dynamics/stability
epitope tags in tandem repeat	FLAG, HA, Myc, Ty1, V5	immunofluorescence, expansion microscopy, immunoprecipitation, western blots
luciferases	Nanoluc, Ppyr9eh	*in vivo* imaging, *in vitro* assays
split proteins	spGFP, spmCherry, spmCherry2, spNG2, spTurboID	studying protein–protein interactions
proximity-dependent biotinylation (BioID)	BirA*, BIOD2, TurboID	identifying transient and stable interactors
fusion tags	mNG::HaloTag, mNG::Ppyr9eh, mNG::BIOID2, mNG::TurboID	combining microscopy with another assay

### 2.2. Brightness of fluorescent protein tags

**Figure 2.**
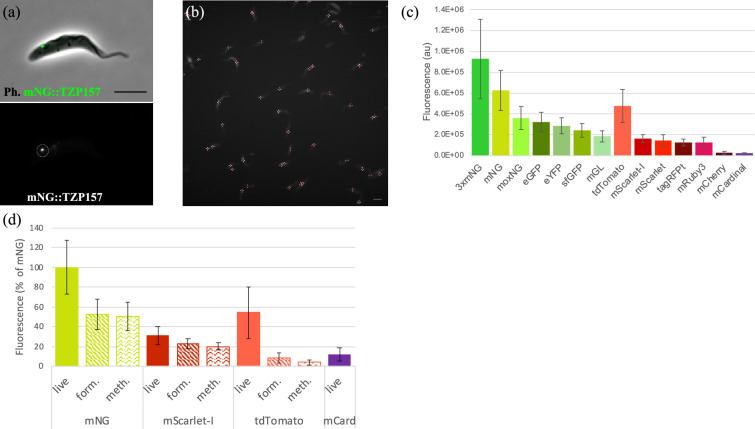
Assessing the brightness of fluorescent proteins tags and their performance in different fixatives. (a) An example cell expressing mNeonGreen-tagged Transition Zone Protein157 (TZP157) at the base of the flagellum. Top panel depicts merged phase contrast and fluorescence; bottom panel shows fluorescence only with the TZP157 punctum to be measured encircled. (b) An example field of cells expressing mNeonGreen-tagged cells with each bright green spot marked for subsequent analysis of its brightness. (c) The brightness of each fluorescent protein was assessed by quantifying the fluorescent signal emanating from the tagged protein. 3×mNG indicates three tandem repeat copies of mNeonGreen and was the brightest tag assessed in this work. (d) The effect of formaldehyde and methanol fixation upon the brightness of mNeonGreen, mScarlet-I and tdTomato was assessed by acquiring a 2 s exposure of fixed versus non-fixed cells and expressed as a percentage mNeonGreen fluorescence from unfixed (live) cells. Error bars = standard deviation; Scale bar = 5 μm; Au = arbitrary units.

**Figure 3.**
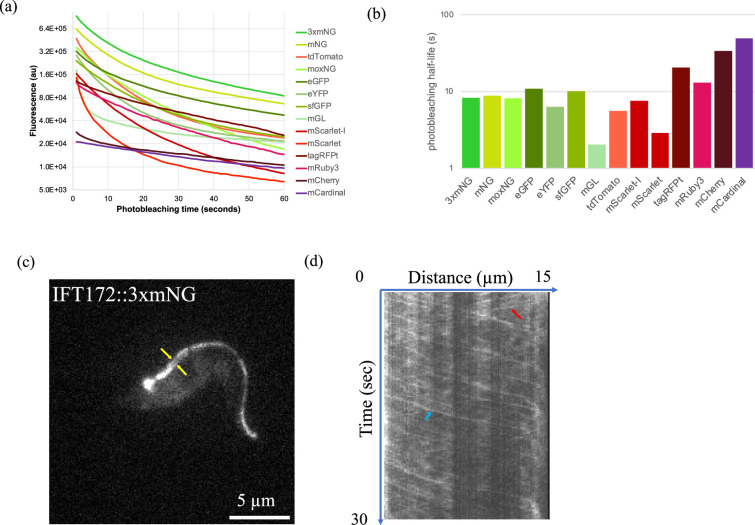
Assessing the photostability of fluorescent protein tags. (a) Cells were irradiated for 30 (1 s) exposures and spots corresponding to tagged transition zone protein 157 were quantified to assess the tag’s photostability. (b) The photostability half-life was calculated by linear interpolation of the two time points that were closest to half maximal brightness. (c) Intraflagellar Transport 172 (IFT172) was tagged on its carboxyl terminus with the triple mNeonGreen tag (IFT::3×mNG). Yellow arrows indicate IFT172 on opposing axonemal doublets. (d) Kymograph analysis demonstrates incorporation of IFT172::3×mNG into moving anterograde (blue arrow) and retrograde (red arrow) trains.

Most fluorescent proteins have available information on their quantum yield, *in vitro* brightness and photostability [11]. However, such information may not reflect their behaviour when used as a protein tag inside a cell due to the tagged protein’s properties and local environment [12]. To assess their *in vivo* utility as protein tags, the flagellar Transition Zone Protein 157kD (TZP157) [13] was tagged on the N terminus using 17 different fluorescent proteins. TZP157 was chosen because it forms a small, single focus inside each trypanosome cell that can be easily quantified using custom scripts (figure 2a, electronic supplementary material, figure S1). The signal intensity in a 1 s exposure was quantified from >100 cells (figure 3b), and it was found that under these conditions, mNeonGreen was the brightest single-copy fluorescent protein tag (figure 3c). A triple mNeonGreen tag, consisting of three copies of mNeonGreen in a tandem repeat, was 50% brighter than a single mNeonGreen. Interestingly, mGreenLantern, which was recently published as a viable alternative to mNeonGreen [5], was the least bright of the green fluorescent proteins tested and highlights that fluorescent proteins behave differently in different contexts. tdTomato [14] was the brightest red fluorescent protein, while mScarlet-I [6] was the brightest monomeric red fluorescent protein. mCherry [14], a widely used red fluorescent protein, did not perform well in this assay and showed significant cellular background (electronic supplementary material, figure S1). The only far-red fluorescent protein tested, mCardinal [15], was faint but detectable, suggesting it as an option for making three-colour trypanosomes or deep-tissue imaging of trypanosomes *in vivo*. Blue fluorescent protein tags did not give a detectable signal at the base of the flagellum (electronic supplementary material, figure S1). To determine whether the high cellular background associated with ultraviolet irradiation prevented their detection, cellular soluble material was extracted using non-ionic detergent to generate cytoskeletons. However, blue-tagged proteins were still not detected, suggesting that they are either too dim or unstable to be useful for most protein tagging in trypanosomes (electronic supplementary material, figure S1).

Based on our experience, the best microscopy data with the fewest artefacts are obtained through live-cell imaging. However, in certain situations, such as performing immunofluorescence analyses, it may be necessary to fix cells prior to imaging. To evaluate the performance of fluorescent protein tags under different fixation conditions, a range of the most promising fluorescent proteins fused to TZP157 was imaged for 2 s after fixation with formaldehyde or methanol and compared with live, unfixed cells (electronic supplementary material, figure S2). Fixation led to a reduction in the brightness of mNeonGreen fluorescence by approximately 50%, but nonetheless, it was comparable with unfixed tdTomato (figure 2d). Surprisingly, formaldehyde fixation resulted in a fivefold reduction in the brightness of tdTomato, and methanol fixation led to a 10-fold reduction. This dramatic decrease in brightness could be attributed to fixatives disrupting the association between individual subunits of the tdTomato dimer. Fixation reduced the brightness of mScarlet-I by only 30–40%, making it a suitable option for imaging fixed red fluorescent proteins. However, the brightness of mCardinal was so greatly reduced by fixation that it was undetectable from cellular autofluorescence (electronic supplementary material, figure S2). Extraction of cytosolic material using detergent revealed a spot at the flagellar transition zone after methanol fixation (electronic supplementary material, figure S2), suggesting that in certain cases mCardinal could be a useful tag even after fixation.

### 2.3. Photostability of fluorescent protein tags

The photostability of fluorescent protein tags plays a critical role in time-lapse experiments, such as the measurement of intraflagellar transport (IFT) rates or the trafficking of secreted cargo. To determine the half-lives of fluorescent proteins under experimentally relevant conditions in live cells, a 30 × 1 s time-lapse movie was conducted on each cell line where a spot corresponding to the tagged TZP157 was visible.

While mCherry and mCardinal were found to be the most photostable, their dimness limits their application in most experimental set-ups (figure 3). In contrast, 3×mNG and mNG were found to exhibit the best combination of brightness and stability, remaining the brightest protein tags throughout the 30 s time-lapse. To test the triple mNeonGreen tag in a dynamic context, we used it to tag a core component of the IFT system, the kinesin–dynein-based transport system that carries components to the flagellar tip [16]. We found that IFT172 tolerated the triple tag well, with cells showing the expected strong fluorescence at the flagellar base and two ‘tracks’ inside the flagellum consistent with transport along opposing axonemal doublets [17]. Kymograph analysis confirmed the presence of anterograde (proximal to distal) and retrograde (distal to proximal) tracks, demonstrating functional incorporation into moving trains (figure 3). Nonetheless, in experiments where the size of the protein tag is a concern, a single mNeonGreen tag may be more suitable. Among the red fluorescent proteins, although tdTomato was found to be the brightest, tagRFPt [18] exhibited the optimal combination of brightness and photostability and was the brightest tag after 24 s of exposure.

### 2.4. mScarlet slow maturation causes an experimental artefact in tagging experiments

The pPOT series, with its inherent diversity, enables the generation of complex cell lines for co-tagging experiments. Using this technique, we tagged two proteins, TZP103.8 and basalin, that localize to the flagellar transition zone at the axonemal base and play crucial roles in nucleating the flagellum central pair of microtubules that coordinate flagellar dynein activity [13,19].

**Figure 4.**
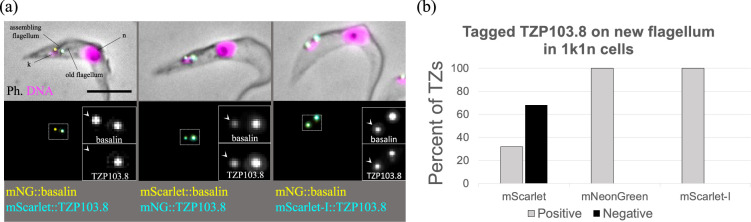
Absence of fluorescent mScarlet::TZP103.8 from assembling flagella is an experimental artefact. (a) Illustrative examples of cells in the very early stages of flagellum assembly with one nucleus (n) and a single, elongated kinetoplast (k), expressing basalin and TZP103.8 tagged with different fluorescent proteins. White arrowheads on the insets indicate the transition zones of assembling flagella in dividing cells; note the absence of mScarlet in the new flagellar transition zone. Scale bar = 5 μm. (b) Cells in the very early stages of flagellar assembly with a single nucleus and kinetoplast (1k1n) were assessed for the presence of tagged TZP103.8 on the new flagellum. When TZP103.8 was tagged with mScarlet, it was not detected in 68% of early new flagella (*n* = 91). In contrast, TZP103.8 is present on all new flagella when tagged with mNeonGreen (*n* = 155) or the fast-folding variant, mScarlet-I (*n* = 94). TZP = transition zone protein.

Our observations indicated that TZP103.8 tagged with mScarlet was evident only in the late stages of flagellum maturation, after the central pair had been assembled and inconsistent with its known role in central pair assembly. Indeed, when examining cytoskeletons in which a new flagellum was visible by phase contrast microscopy but the mitochondrial DNA (kinetoplast) and nucleus had not yet segregated (termed ‘1k1n’ cells, figure 4a), we noted that more than 75% of the assembling flagella were negative for mScarlet-tagged TZP103.8 (figure 4b).

To investigate this further, we swapped the tags, tagging TZP103.8 with mNeonGreen and basalin with mScarlet. With this change, all new flagella were positive for tagged TZP103.8. A review of the literature suggested that the slow maturation of mScarlet may have caused this apparent absence [6,12,20]. To test this hypothesis, we employed mScarlet-I, a fast-folding variant of mScarlet [6,21] that differs in a single amino acid change proximal to the chromophore. When we used mScarlet-I as a tag, TZP103.8 was detectable at the very earliest stages of flagellum assembly, consistent with mNeonGreen (figure 4).

Our findings indicate that mScarlet-I is a more suitable protein tag in most experimental set-ups, and that caution should be exercised when using fluorescent proteins as protein tags. Nevertheless, the delayed onset of mScarlet fluorescence may serve as a ‘molecular timer’ to determine the relative age of different cellular pools of protein.

### 2.5. Epitope tags for immunofluorescence and expansion microscopy

Expansion microscopy is an emerging super-resolution technique that combines immunofluorescence with physical expansion of the sample to obtain high-resolution localization information [8,9,22]. To facilitate the adoption of expansion microscopy in trypanosomatid research, we developed a set of pPOT tagging plasmids encoding epitopes (Ty1, HA, Myc, FLAG and V5) in tandem repeat (table 1).

**Figure 5.**
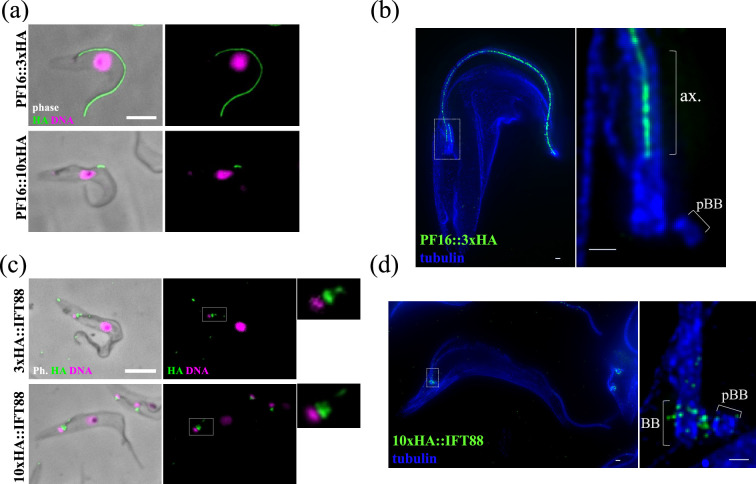
Deca-repeat epitopes enhance signal strength, while triple repeats minimize localization interference. (a) PF16 was tagged at the C-terminus with either three or 10 copies of the HA epitope tag, and cytoskeletons were analysed by immunofluorescence. Cells expressing PF16::10HA exhibited patchy or absent flagellar fluorescence, indicating incorrect incorporation of the tagged protein. In contrast, PF16::3HA was correctly incorporated along the entire length of the axoneme. (b) Expansion microscopy analysis of cells expressing PF16::3 HA shows strong signal at the centre of the axoneme lumen; left panel is a 13-slice *Z* projection of an entire cell; right is an enlarged inset showing a single slice of an individual axoneme base. (c) IFT88 was tagged at the N-terminus with either three or 10 copies of the HA epitope tag, and cytoskeletons were analysed by immunofluorescence; although both tandem epitopes gave rise to the same localization, the 10× HA gave a significantly stronger signal (approx. sixfold in this example). (d) Expansion microscopy analysis of 10×HA::IFT88 expressing cells shows a localization consistent with ninefold symmetry at the transitional fibres encircling the flagellar base: left panel is a projection of an entire cell; right panel shows the basal body projection only. ax = axoneme, pBB= pro-basal body, BB = basal body. Scale bar in (a,b) = 5 μm, in (c,d) = 1μm.

To evaluate these constructs, we first tagged PF16, a well-characterized component of the flagellar central pair apparatus (CPA) that runs through the axoneme lumen [23]. Immunofluorescence analysis of PF16 tagged with 10 copies of the HA epitope resulted in either absent or 'patchy' axoneme localization (figure 5), suggesting that the deca-tag had disrupted incorporation into the CPA, possibly by interfering with polymerization of PF16 dimers [24]. This was consistent with several other deca-tags available with the pPOT series (data not shown). To address this issue, we made a pPOT plasmid encoding triple-HA tandem epitopes; this was well-tolerated and immunofluorescence analysis yielded the expected unbroken flagellar axoneme signal. Despite the reduced number of epitopes, expansion microscopy analysis of PF16::3xHA expressing cells produced a strong and specific signal in the axoneme centre emanating from its base, consistent with correct central pair localization.

In contrast, both the triple tag and the deca-tag gave the expected localization when used to tag IFT88 (figure 5). As anticipated, in detergent-treated 'cytoskeletons’, most flagellar IFT material was extracted, leaving the more strongly associated IFT material at the flagellar base. The deca-tag provided a much brighter signal than the triple-tag, consistent with increased stoichiometry of antibody binding sites in the larger tandem epitope. Expansion microscopy analysis of 10×HA::IFT88 expressing cells revealed punctate signal encircling the flagellar basal body, consistent with accumulation at the tips of the transitional fibres [25].

We expect the deca-epitope tags to provide the highest quality data in most cases due to increased signal. However, the triple epitope tag may be more suitable in some contexts, particularly when protein function might be compromised by larger tags.

### 2.6. Tagging *Leishmania mexicana* proteins

**Figure 6.**
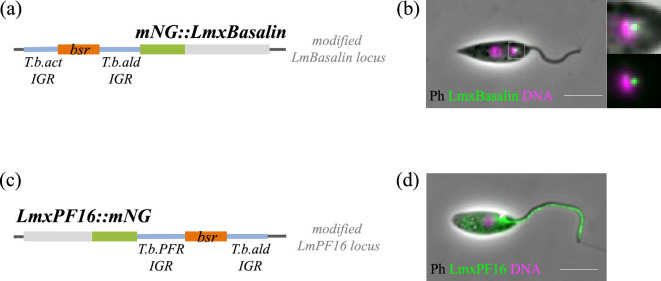
pPOT tagging plasmids are functional in *L. mexicana*. (a) Schematic of the LmxBasalin locus when modified with the pPOTv7-blast-mNG N-terminal tagging amplicon. (b) Live-cell fluorescence microscopy of LmxBasalin tagged on the N terminus using the trypanosome pPOT plasmid reveals a bright spot at the flagellar base consistent with the predicted flagellar transition zone localization. (c) A schematic of the LmxPF16 locus when modified with the pPOTv7-blast-mNG C terminal tagging amplicon. (d) Live-cell fluorescent microscopy of LmxPF16 tagged on the C terminus using the trypanosome tagging plasmid reveals a strong axoneme signal consistent with correct localization to the flagellar central pair.

Kinetoplastids appear to accept foreign regulatory nucleotide sequences [26], suggesting that the pPOT tagging amplicons may be functional in trypanosomatids other than *T. brucei*. To test this, we set out to tag two different *L. mexicana* proteins with mNeonGreen using a pPOT tagging plasmid: LmxBasalin on the N terminus and LmxPF16 on the C terminus (figure 6). In both cases, drug-resistant clones were successfully selected, demonstrating that the *T. brucei* regulatory sequences flanking the drug resistance cassettes were functional in *L. mexicana*.

Importantly, fluorescent protein localization produced the expected results (figure 6). mNG::LmxBasalin generated a strong fluorescent focus at the flagellar base, consistent with its established flagellar transition zone localization [19]. Similarly, LmxPF16::mNG produced a linear signal along the length of the flagellar axoneme, consistent with correct localization to the central pair. Some accumulation of fluorescence at the flagellar base was observed, possibly indicating a ‘staging post’ prior to flagellar import, or perhaps aberrant accumulation due to the mNeonGreen tag. Therefore, our data clearly demonstrate that the pPOT *T. brucei* genetic regulatory sequences are functional in *L. mexicana* and likely in other trypanosomatids.

### 2.7. Tagging signal peptide and glycosylphosphatidylinositol-anchored proteins

GPI-anchored proteins are characterized by an N-terminal signal peptide that guides them to the endoplasmic reticulum and a GPI-anchor addition site near the C-terminus that secures the mature protein to the outer leaflet of the surface membrane. Due to the cleavage of both N and C termini during protein processing, GPI-anchored proteins are not suitable for tagging using conventional pPOT plasmids.

**Figure 7.**
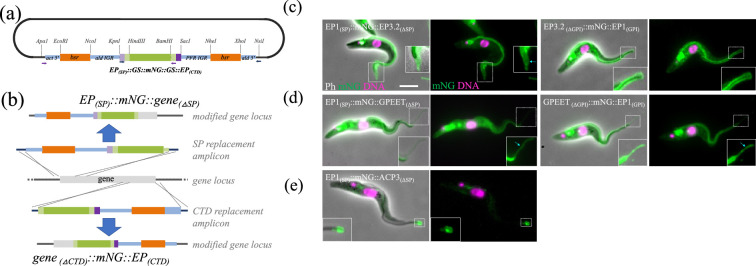
Tagging GPI-anchored and signal peptide surface proteins. (a) pPOTvGPI(EP)-blast-mNG was developed to tag proteins with predicted GPI anchor addition and/or N-terminal signal peptides. Sequence encoding mNeonGreen was flanked by EP1 processing signals. (b) PCR amplicons derived from pPOTvGPI(EP)-blast-mNG are targeted to replace sequence encoding either the signal peptide (SP), or the C terminal GPI-anchor addition and cleavage sites (CTD), with mNeonGreen fused to the equivalent sequence from EP1 procyclin (Tb927.10.10260: amino acids 1−30 or 117−141 for signal peptide replacement or carboxyl terminal domain (CTD) replacement, respectively). (c−e) Live-cell fluorescence microscopy of cells expressing tagged GPI-anchor proteins. (c) EP3.2 procyclin (Tb927.6.520: amino acids 1−27 or 107−129 replaced), (d) GPEET (Tb927.6.520: amino acids 1−20 or 92−114 replaced), (e) ACP3 (Tb927.7.7470: amino acids 1−16 replaced). Inset in (e) highlights the flagellar tip enrichment of ACP3. Insets are displayed at high-contrast levels to reveal fluorescent tubules and blebs (blue arrows) that indicate membrane association of the chimeric protein. Scale bar = 5 μm.

Inspired by previous research [27], we developed a new pPOT plasmid called pPOTvGPI(EP). This plasmid encodes an mNeonGreen tag flanked by the signal peptide and GPI-anchor processing signals of EP1 procyclin, a major surface protein found in insect midgut form trypanosomes (figure 7) [28]. We selected mNeonGreen as the fluorescent marker due to its rapid folding properties [4] and extensive validation in genome-wide protein tagging, which included numerous membrane-associated proteins trafficked through the endoplasmic reticulum (ER) [29].

Through careful design of the PCR-tagging primers, the signal peptide of the target protein can be replaced with the EP1 signal peptide fused to mNeonGreen (EP_(SP)_::mNG). Similarly, the C-terminal domain of the target protein containing the GPI-anchor addition and cleavage sites can be replaced with mNeonGreen fused to EP1 procyclin’s C-terminal GPI-anchor processing signals (mNG::EP_(CTD)_). In both cases, the mNeonGreen fluorescent protein is expected to form part of the mature, processed protein, allowing determination of its localization.

To evaluate this new tagging plasmid, we targeted two different GPI-anchored proteins (EP3-2 procyclin [28] and GPEET [30]), and one protein with an N-terminal signal peptide (ACP3 [31]), and determined the localization of the tagged protein (figure 7). In some instances, we noted enrichment in the endosome or the ER indicating accumulation at particular stages of trafficking, indicating either a genuine trafficking staging post or inefficient processing of the chimeric protein by the cellular machinery. Nonetheless, in all cases we successfully reproduced the expected surface localizations. Hence, tagged EP3-2 and GPEET were found to localize correctly to the cell body and flagellar surface membranes, while tagged ACP3 concentrated at the membrane of the flagellar tip.

## 3. Discussion

The pPOT plasmids described here signify a notable improvement in the previous generation of pPOT plasmids [3]. These new plasmids have a range of tags and drug resistances that allow for the creation of cell lines with multiple different tagged proteins, enabling co-localization and biochemistry studies to be conducted. Moreover, the use of universal primer annealing sequences considerably reduces the cost and enhances the flexibility of the system because a single long primer pair can be used to tag a target protein using a variety of protein tags or drug resistances.

The modularity of these plasmids allows for the easy incorporation of new primary tags and flanking epitopes into the toolkit as improved biochemical or fluorescent tags become available. Additionally, the development of modular ‘fusion tags’ makes it possible to tag proteins with tags that have multiple functions. This enables the combination of, for instance, bioluminescence and fluorescence imaging, as elegantly demonstrated in *Trypanosoma cruzi* [32], to locate parasites more easily in infected tissue. Similarly, other fusion tags described here, which combine fluorescent tags with proximity-dependent biotinylation, enable the confirmation of localization and the subsequent identification of interacting proteins. The development of a set of plasmids that incorporate either 10 or three tandem copies of five different epitope tags supports the emerging technique of expansion microscopy because it facilitates tagging of multiple proteins for ultrastructural protein cartography. Moreover, although not tested here, we anticipate that the availability of split fluorescent protein tags, click chemistry (HaloTag, CLIP and SNAP tags) and proximity-dependent biotinylation (BirA*, TurboID and BIOID2) tags will enable easy and scalable protein–protein interaction studies.

Although in our laboratory, we have successfully made dozens of different co-tagged cell lines expressing up to four different tagged proteins for various analyses, we note that, on occasion, it was necessary to screen multiple clones before identifying a correctly co-tagged clone. We have not investigated this further, but suspect that it is due to tagging amplicons mis-integrating into previously tagged loci due to the high degree of homology between different tagging amplicons. Nonetheless, once a correctly co-tagged clone was identified, it remained stable even in long-term culture.

We tested 17 different fluorescent proteins in microscopy imaging and showed that mNeonGreen remains the brightest fluorescent protein even after extended illumination and fixation, making it the best choice in most applications. This is supported by its success in the TrypTag whole genome tagging project [29,33] and in tagging a variety of cytological markers [34]. We also observed a putative ‘slow folding’ artefact of mScarlet, meaning that, for most purposes, the fast-folding variant mScarlet-I is the preferred red fluorescent protein. However, for time-lapse imaging, tagRFPt would be the preferred red fluorescent protein, as it performs well over a 30 s time-lapse movie. High-intensity irradiation using a metal-halide lamp and standard filter sets were used in this study. Optimization of such parameters would likely improve performance. Nonetheless, our data underscore that the behaviour of fluorescent proteins differs as protein tags inside a living cell from that predicted by their *in vitro* determined characteristics, such as quantum yield. Moreover, it is likely that fluorescent proteins perform differently in different cell types or different cellular locations [12]. Therefore, although TZP157 tagging data provided here provide a baseline, testing protein tags in a realistic setting is essential.

We found that, although deca-tags gave the brightest signal, in some cases they interfered with localization, and presumably function, of the tagged protein. In the case of PF16, this was overcome using the smaller triple HA tag. Given that PF16 was successfully tagged with the larger mNeonGreen (28.5 kD) by multiple studies, this is unlikely to be due solely to the molecular weight of the protein tag. Although the isoelectric points (pI) of the deca-HA and the triple HA are rather similar (3.04 versus 3.57, respectively), it may be that the larger deca-tag confers a stronger localized charge change that disrupts oligomerization of the more neutral PF16 dimer (pI 5.56) via electrostatic repulsion. This highlights the importance of having a range of tags with different biochemical properties to match proteins of interest (see electronic supplementary material, file S5 for the molecular weights and isoelectric points of the pPOT tags). For example, basic proteins, such as histones, might be best tagged with similarly basic tags to avoid modifying their *in vivo* interactions and function. The general point to be made is that one should always consider the potential influence of tag size and pI on the resulting chimeric version of the protein of interest.

Despite using *T. brucei* regulatory sequences, the pPOT plasmids work in *L. mexicana* and probably other trypanosomatids, such as *T. cruzi* and *Trypanosoma congolense*. Given that the pLENT, pNUS and pPLOT series expression plasmids use *Crithidia fasciculata* intergenic sequences, this was not unexpected and, in fact, the incorporation of exogenous DNA might even offer an advantage by reducing interference with the organism’s endogenous gene regulation. This significantly increases the value of the pPOT plasmid series and complements the excellent genetic tools already available in these systems [26,32,35].

GPI-anchored and signal peptide proteins have important roles in host–parasite interaction and are key virulence factors, representing dominant components of the trypanosome surface coat. We demonstrate the ability to easily tag such proteins by replacing sequences at the amino or carboxyl terminus with the corresponding sequence of EP1 procyclin fused to mNeonGreen. Although there were minor trafficking variations, we were able to reproduce the target protein’s localization, indicating that the exogenous trafficking signals of the chimeric protein did not dominate. Nonetheless, caution should be exercised when interpreting the behaviour of chimeric proteins and it is not clear whether the GPI-protein tagging plasmid we describe here would work in bloodstream form trypanosomes or related trypanosomatids. However, the modularity of the GPI-anchor tagging plasmid supports easy replacement of the EP1 encoding sequence with the target protein’s processing signals in circumstances where this is a concern. Therefore, this approach opens new avenues for studying the dynamics and functions of surface proteins and represents a valuable addition to the trypanosomatid research toolkit.

We anticipate that this extensive set of more than 100 pPOT plasmids will be an important toolset for investigating trypanosomatid protein localization and function.

## 4. Material and methods

### 4.1. Fluorescent protein microscopy

Images were acquired using a Leica DM6 B microscope equipped with a metal halide lamp (EL 6000, 11504115) serving as the illumination source, a 63× objective with a numerical aperture of 1.30 (11506385) and a Leica K5 Microscope Camera (11547112). Filters used for fluorescence imaging depended on the spectral properties of the fluorescent proteins: blue fluorescent proteins: DAPI ET, 11504203; green fluorescent proteins: GFP ET, 11504164; red fluorescent proteins: RHOD ET, 11504205; and far-red fluorescent protein: Y5 ET, 11504171. Samples were prepared for live-cell and fixed-cell imaging as described [25]. Briefly, cells were washed and settled on clean glass slides prior to imaging (live-cell) or fixation. Cells were either fixed in cold methanol or 2% formaldehyde for 10 min, and fixed cells were mounted in phosphate-buffered glycerol containing 1,4-diazabicyclo[2.2.2]octane (DABCO) and imaged immediately. Data for calculating brightness and photostability of live cells were acquired by performing a 30 × 1 s time lapse, with the first image in the series being used to calculate brightness, and the entire series being used to calculate photostability. Data to calculate the brightness of fluorescent proteins under different fixation conditions were acquired by performing a single 2 s exposure.

### 4.2. Fluorescent protein brightness and photostability analyses

Spots corresponding to tagged TZP157 were initially identified using FIJI’s built-in ‘Find Maxima’ and then fields were manually curated. Brightness of each spot was quantified using a custom FIJI script (electronic supplementary material, files S1 and S2). Briefly, a square was drawn around each maxima and the signal intensity measured, and the local background subtracted using the median pixel value of a larger square centred on the TZP157 spot. This was repeated on each spot corresponding to tagged TZP157 at each timepoint, with more than 100 cells analysed per timepoint. The photostability half-life of each protein was determined by calculating the linear interpolation between the two time points that were closest to half the brightness value after 1 s.

### 4.3. Immunofluorescence and expansion microscopy

For immunofluorescence, non-ionic detergent-treated ‘cytoskeletons’ were prepared, fixed in methanol and rehydrated in phosphate-buffered saline (PBS) as described [36]. Slides were then incubated in block (2% fetal calf serum, 2% TWEEN, in PBS) for 30 min, stained with anti-HA (AF291, ABCD antibodies) diluted 1/500 in block for 1 h, washed in PBS and stained with secondary antibody (A48279, Invitrogen) diluted 1/1000 in block for 1 h, prior to mounting in phosphate-buffered glycerol and imaging. Expansion microscopy was performed on cytoskeletons as described [8]. Briefly, cytoskeletons were incubated in fixative (0.7% formaldehyde and 1% acrylamide in PBS) overnight at room temperature in the dark and gelated in a monomer solution (19% sodium acrylate, 10% acrylamide, 0.1% 2% N,N′-methylenebisacrylamide, 0.5% N,N,N′,N′ -tetramethylethylenediamine and 0.5% ammonium persulphate in PBS) for 5 min on ice. After a 30 min incubation at 37°C, gels were heated in denaturation buffer (50 mM Tris, 200 mM NaCl_2_, 200 mM sodium dodecyl sulfate and pH9) for 90 min at 98°C and then expanded using three washes in excess water. The gel was incubated in block for 30 min and stained with anti-HA antibody (AF291, ABCD antibodies) diluted 1/500 in block overnight at room temperature. After two washes in water, the gel was stained in secondary antibody diluted 1/1000 in block for 6 h. The gel was then counter-stained with anti-alpha tubulin (ABCD_AA345) and matching secondary antibody (anti-guineapig-555, A21435, Invitrogen), and imaged using a Leica DMi8 fluorescence microscope. Data were deconvolved using the DeconvolutionLab2 plugin (v. 2.1.2) [37] for Fiji (v. 1.54 f) [38] using the Richardson–Lucy algorithm with 30 iterations. Synthetic point spread functions were used and generated with the PSF Generator plugin for Fiji [39]. The Born and Wolf 3D Optical Model was used with parameters: refractive index: 1.518; accuracy computation: good; wavelength: 488 and 555; NA: 1.32; pixel size *XY*: 65 nm; pixel size *Z*: 220 nm; size *XYZ*: 64 × 64 × 8.

### 4.4. Plasmids and cell lines

Modules were cloned into the pPOT plasmid using standard molecular biology techniques. Where necessary, genes were synthesized using either Twist Bioscience or Life technologies, with custom scripts performing codon optimization based on the codon frequency of the trypanosome genome. Primers for *T. brucei* gene tagging were designed using TagIt [3], and primer designs for tagging every trypanosome gene in the 927 and 427 reference genomes are provided in electronic supplementary material, file S3 as a community resource. Primers for *L. mexicana* gene tagging were designed using LeishGEdit (http://www.leishgedit.net/Home.html [26]). Amplicons and transgenic cell lines were produced as described [3,26]. Primers to tag predicted GPI-anchored proteins were designed using a custom Python script (electronic supplementary material, file S4) based on Signal-P [40] (to predict the signal peptide) and NetGPI [41] (to predict the GPI-anchor addition and cleavage sites). Procyclic form *T. brucei* TREU927 and promastigote form *L. mexicana* MHOM/GT/2001 /U1103 reference genome cells were used for all experiments.

## Data Availability

All plasmids and plasmid sequences are available from Addgene. Supplementary material is available online [42].
